# Relationship of the anterior ethmoid sinus to the lacrimal sac: a computed tomography study

**DOI:** 10.1016/j.bjorl.2022.02.002

**Published:** 2022-02-28

**Authors:** Beatriz de Costa Remor, Leonardo Balsalobre, Simone Haber Duellberg Von Faber Bison, Maria Helena Salgado Delamain Pupo Nogueira, Ana Taíse de Oliveira Meurer, David Caetano Bastos, Aldo Cassol Stamm

**Affiliations:** a Hospital Edmundo Vasconcelos, Centro de Otorrinolaringologia e Fonoaudiologia, São Paulo, SP, Brazil; b Hospital Albert Einstein, São Paulo, SP, Brazil; c Hospital São Paulo, São Paulo, SP, Brazil

**Keywords:** Dacryocystorhinostomy, Agger nasi, Anterior ethmoid sinus, Ethmoidal sinus, Computed tomography

## Abstract

**Objective:**

To analyze the anatomical relationship between the lacrimal sac and the agger nasi cell on Computed Tomography (CT); to correlate the right and left sides on each scan.

**Methods:**

CT scans of adult patients were reviewed for pneumatization of the agger nasi and its relationship to the lacrimal sac. The degree of agreement between the right and left sides was also evaluated.

**Results:**

A total of 130 CT scans were examined. An agger nasi cell was found medial to the lacrimal sac in 59.23% of scans. On 86.15% of scans, pneumatization was similar on both sides.

**Conclusion:**

The agger nasi air cell is located medial to the lacrimal sac in more than half of individuals. The right and left sides exhibit the same pneumatization pattern in approximately 80% of cases.

**Level of evidence:**

4.

## Highlights

• The agger nasi is medial to the lacrimal sac in most patients.
• The agger nasi can be considered an anatomical key point in endoscopic DCR.
• The right and left sides of each patient have similar anatomy.

## Introduction

The nasolacrimal drainage system has a direct anatomical relationship with the lateral wall of the nose. The success of Dacryocystorhinostomy (DCR), whether external or endoscopic, requires in-depth knowledge of this region by the surgeon.

The anatomical boundaries of the nasolacrimal system include the anterior ethmoid cells, the frontal process of the maxilla, the inferior turbinate, and the lacrimal bone proper. The key to surgical success in DCR is wide exposure of the proximal part of the lacrimal sac, at the level of the common canaliculus and lacrimal sac fundus.^1^, ^2^, ^3^

Studies support that the lacrimal sac should be opened as widely as possible during surgery to achieve the best outcome in endoscopic DCR.^4^, ^5^, ^6^ The most common cause of unsuccessful surgery is operator error when siting and sizing the drainage ostium fashioned in the bone of the lateral wall of the nose.^7^

The lacrimal drainage system has a very close anatomical relationship to the anterior ethmoid sinus, and the relationship between the lacrimal sac fundus and the agger nasi air cell (the anteriormost cell of the ethmoid sinus) is well known.^1^ Intraoperatively, the Agger Nasi (AN) is often found medial to the lacrimal sac. This cell needs to be removed to achieve proper access to the lacrimal sac.^8^ However, this anatomical relationship has been poorly studied on Computed Tomography (CT) scans, especially considering the close correlation between the lacrimal sac and the agger nasi and its consequent influence on DCR outcomes.^9^

## Methods

This is a retrospective study in which CT scans of the paranasal sinuses were analyzed using the electronic PACS system. All CT scans performed from January through August 2020 were analyzed.

All CT scans were noncontract, performed using the same system (Optima CT660), with a slice thickness of 0.625 mm. Multiplanar Reformatting (MPR) was performed in the axial, coronal, and sagittal planes of all images. Patients who had a history of previous sinonasal surgery, sinonasal tumors, or facial trauma were excluded, as were those younger than 14 years.

**Figure 1:**
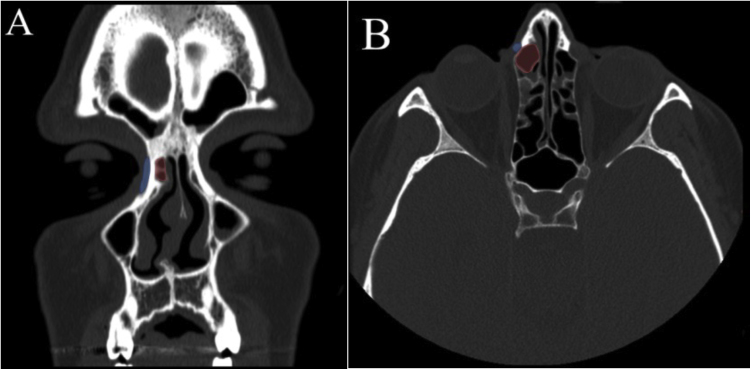
Agger nasi cell located medial to the lacrimal sac. (A) CT scan (coronal slice) showing agger nasi cell (red) medial to the lacrimal sac (blue) at the level of the common canaliculus on the right. (B) CT scan (axial slice) showing agger nasi cell medial to the lacrimal sac on the right.

Axial slices were examined for the presence of an agger nasi air cell medial to the lacrimal sac at the level of the common canaliculus, i.e., 5 mm below the lacrimal sac fundus. This position was found by measuring the lacrimal sac fundus in the coronal plane (Fig. 1).^1^ Areas 2 mm above and below this location were also examined to confirm if the agger nasi cell was located medial to the lacrimal sac on its medial wall.

**Figure 2:**
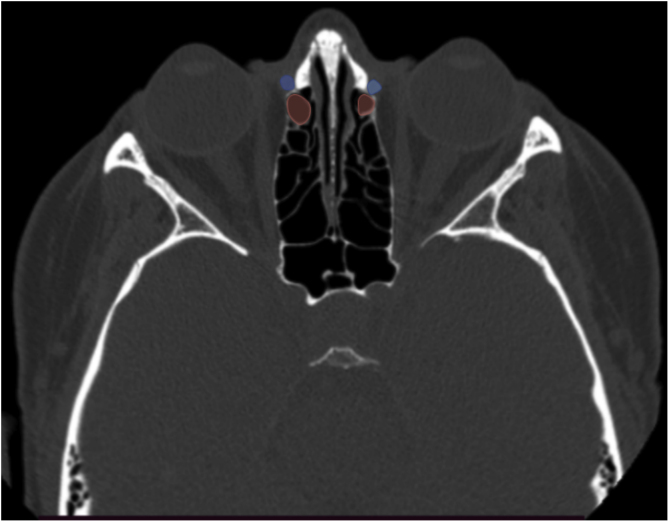
CT scan (axial slice) showing agger nasi cell (red) located posterior to the lacrimal sac (blue) bilateral.

The statistics were calculated considering whether or not the AN cell was in contact with the medial boundary of the lacrimal sac, i.e., if the AN cell was located medial to the lacrimal sac. Cases in which the agger nasi did not contact the medial wall of the lacrimal sac were recorded as “no” (Fig. 2). The degree of agreement between the right and left sides on each scan was also assessed.

The research was submitted and approved by the Research Ethics Committee under the number 4,677,941.

## Results

A total of 132 CT scans were analyzed. Two patients were excluded due to distorted anatomy as a result of disease or previous surgery. Overall, 260 sides (from 130 CT scans) were examined for statistical analysis. Patients’ ages ranged from 14 to 77 years (mean, 36 years). Most patients (50.76% of the sample) were female. The main indication for CT in these patients was evaluation of nasal complaints by the ENT team. The conditions detected on included CT scans either did not have direct contact with or did not distort the nasolacrimal system or the anterior ethmoid sinus.

An agger nasi cell was found medial to the lacrimal sac in 154 sides, or 59.23% of the sample (Table 1). The results of correlation analysis between the right and left sides are shown in Table 2. Overall, 86.15% of sides exhibited concordant pneumatization.

**Table 1:** Agger nasi cell located medial to the lacrimal sac on the right or left (yes or no).

	Right side	Left side	Overall
**Yes**	79	75	154 (59.23%)
**No**	51	55	106 (40.76%)
**Overall**	130	130	260 (100%)

**Table 2:** Correlation between the right and left sides.

AN medial to lacrimal sac bilaterally	AN not medial to lacrimal sac bilaterally	AN medial to lacrimal sac unilaterally	Overall
68 (52.30%)	44 (33.84%)	18 (13.84%)	130 (100%)

## Discussion

Anatomical and CT studies shows that the agger nasi cell is present in 80% to 98.5% of individuals.^8^, ^10^, ^11^ Previous descriptions suggest that, in 86% of individuals, this cell can extend anteriorly to the suture of the lacrimal bone and maxilla. On the other hand, in 41% to 90% of individuals, the agger nasi cell may present anterior to the posterior lacrimal crest, i.e., lying medial to the lacrimal bone.^7^, ^8^, ^12^, ^13^, ^14^

The main causes of endoscopic DCR failure have been attributed to difficulty in locating the lacrimal sac, insufficient osteotomy, inadequate opening of the sac, granulation tissue, fibrosis, and new bone formation.^15^ The success of this surgical procedure depends on locating the sac precisely, creating an osteotomy large enough to expose the entire sac, and achieving complete marsupialization of the sac.^16^

It has been well demonstrated in the literature that positive results (and long-term outcomes) of DCR depend on the extent and location of the opening of the lacrimal system into the nasal cavity,^2^ extending from the lacrimal sac fundus to the region superior to the axilla of the middle turbinate;^17^ ensuring that the ostium is created at the level of the common canaliculus; and marsupialization and integration of the lacrimal sac into the lateral nasal wall.

As pneumatization of the agger nasi cell is closely related to the medial surface of the lacrimal sac, this structure can obstruct the surgeon’s access towards the lacrimal fossa during endoscopic surgery or may even be confused with the lacrimal sac. The literature confirms that, in 55% of patients, the agger nasi cell can be found at a level that could hinder endoscopic DCR^8^ and necessitate its removal. According to the data found in our sample, the agger nasi would not require opening in all patients, as it was medial to the lacrimal sac in 59.23% of cases. However, finding the agger nasi and even removing its anterior wall can serve as an important point of anatomical reference during surgery, and can be considered a posterior landmark for opening of the lacrimal sac, thus ensuring a wide enough opening of the sac into the nasal cavity at the level of the common canaliculus, thus reducing the risk of failure to locate and open the lacrimal sac.

Most of the analyzed CT scans (86.15%) showed concordant anatomy when comparing the right and left sides, i.e., if the agger nasi is pneumatized medial to the lacrimal sac on one side, it will likely be so on the contralateral side. This can be very useful information to the surgeon in bilateral procedures, due to the anatomical similarity of both sides.

## Conclusion

The agger nasi air cell was located medial to the lacrimal sac in 59.23% of cases in our sample. Pneumatization of the agger nasi was concordant between the right and left sides in 86.15% of patients.

## Conflicts of interest

The authors declare no conflicts of interest.
